# Impact of the kidney transplantation moratorium in France because of the COVID-19 pandemic

**DOI:** 10.1093/eurpub/ckac130.224

**Published:** 2022-10-25

**Authors:** V Bonnemains, F Le Borgne, E Savoye, C Legeai, M Pastural, S Bayat-Maokei, R Lenain, K Leffondré, C Couchoud, Y Foucher

**Affiliations:** 1 INSERM UMR 1246 - SPHERE, Université de Nantes, Université de Tours, Nantes, France; 2 IDBC-A2COM, Nantes, France; 3 Agence de la Biomédecine, Saint-Denis, France; 4 Université de Rennes, EHESP, CNRS, INSERM, Arènes – UMR 6051, U1309, Rennes, France; 5 Service de Néphrologie, Centre Hospitalier Universitaire de Lille, Lille, France; 6 Université de Bordeaux, INSERM, UMR1219, Bordeaux, France; 7 Université de Lyon I, CNRS, UMR 5558, Villeurbanne, France; 8 Centre Hospitalier Universitaire de Nantes, Nantes, France

## Abstract

**Background:**

The COVID-19 pandemic has resulted in worldwide kidney transplantation (KT) moratoriums. The impacts of these moratoriums on the life expectancy of KT candidates remain unclear.

**Methods:**

We simulated the evolution of several French candidate populations for KT using a multistate semi-Markovian approach and according to moratorium durations ranging from 0 to 24 months. The transition rates were modeled from the 63,927 French patients who began dialysis or were registered on the waiting list for KT between 2011 and 2019.

**Results:**

Among the 8,350 patients active on the waiting list at the time of the French KT moratorium decided on March 16, 2020, for 2.5-months, we predicted 4.0 additional months [CI: 2.8, 5.0] on the waiting list and 42 additional deaths [CI: -70, 150] up to March 16, 2030. In this population, we reported a significant impact for a 9-month moratorium duration: 135 attributable deaths [CI: 31, 257] up to March 16, 2030. Patients who became active on the list after March 2020 were less impacted.

**Interpretation:**

The temporary moratorium of KT during a COVID-19 peak in order to free up hospitals’ resources doesn't impact patients’ 10-year survival if the moratorium does not exceed a prolonged period.

Funding. French National Research Agency (ANR-20-COV8-0002-01).

**Key messages:**

- The French 2020 KT moratorium didn't impair patients’ 10-years survival.
- 9-month or longer moratoriums may impair patients’ 10-years survival.

